# Does Early Graft Patency Benefit from Perioperative Statin Therapy? A Propensity Score-Matched Study of Patients Undergoing Off-Pump Coronary Artery Bypass Surgery

**DOI:** 10.1155/2019/1582183

**Published:** 2019-08-06

**Authors:** Shanglin Chen, Hengchao Wu, Tao Yang, Baotong Li, Yuanyu Hu, Hansong Sun

**Affiliations:** Department of Cardiac Surgery, Fuwai Hospital, National Center for Cardiovascular Diseases, Chinese Academy of Medical Sciences and Peking Union Medical College, Beijing 100037, China

## Abstract

**Background:**

Decreased graft patency after off-pump coronary artery bypass grafting (OPCAB) leads to substantial increases in cardiac events. However, there is paucity of data on efficacy and safety of perioperative statin therapy for OPCAB populations.

**Methods:**

582 patients undergoing OPCAB in a single-institution database (October 1, 2009–September 30, 2012) were stratified by perioperative continuation of statin therapy (CS group, n=398) or not (DS group, n=184). Inverse probability weighted propensity adjustment was used to account for treatment assignment bias, resulting in a well-matched cohort. Primary outcomes were graft patency at an average of five days after operation and in-hospital mortality. Secondary outcomes included intraoperative blood loss, liver, and renal functions.

**Results:**

No in-hospital death occurred in this study. Early graft patency rates after OPCAB were 98.4% (1255 of 1275 grafts) in the CS group and 98.0% (583 of 595 grafts,* P*=0.486) in the DS group. Secondary outcomes showed a reduction in blood loss during operation (438.53 mL versus 480.47 mL,* P*=0.01). Continuation of statin therapy is associated with alanine transaminase (ALT) elevation (49.67 U/L versus 34.52 U/L,* P*<0.001), as well as aspartate transaminase (33.54 U/L versus 28.10 U/L,* P*<0.001). Abnormal ALT elevation was observed in 8.9% of the CS group and 3.1% in DS (odds ratio 3.06, 95% confidence interval, 1.77 to 5.29,* P*<0.001). There was no significant difference in estimated glomerular filtration rate (76.28 mL/min/1.73m^2^ versus 76.13 mL/min/1.73m^2^,* P=*0.90). Subgroup analyses suggested that graft occlusion was less common in CS than in DS group among smoking patients (odds ratio 0.41, 95% confidence interval, 0.20 to 0.86,* P*=0.026).

**Conclusions:**

Perioperative continuation of statin therapy did not improve early graft patency in OPCAB patients. A lower risk of graft occlusion was observed among smoking patients. Continuous statin use correlated with liver function elevation (Clinical Trials.gov number, NCT 01268917).

## 1. Introduction

In 2017, European Association for Cardiothoracic Surgery (EACTS) guidelines on perioperative medication in adult cardiac surgery suggested that it was not recommended to initiate statin therapy shortly before cardiac surgery [1].

Off-pump coronary artery bypass grafting surgery (OPCAB) has offered a promising alternative strategy for revascularization. Undoubtedly, graft patency is one of the most widely studied areas among these populations because of its significant role in long-term outcomes and quality of life [2–4], as OPCAB is technically more demanding than on-pump coronary artery bypass surgery regarding the operative field, target vessels, and learning curve for the procedure [5, 6].

Chronic statin therapy has an established role on delaying atherosclerosis progression in native coronary arteries and reducing adverse cardiovascular events among CABG patients [7–9]. However, the efficacy and safety of perioperative statin therapy still remain controversial [2, 10–12].

To evaluate the efficacy and safety of perioperative continuation of statin therapy in patients undergoing OPCAB, this retrospective cohort study was conducted to determine the impact of perioperative statin use on early graft patency as well as other in-hospital outcomes.

## 2. Methods

### 2.1. Patient Population and Definition

Five hundred eighty-two patients underwent first-time isolated OPCAB, with continuation or discontinuation of statin therapy in the preoperative period and after surgery, Fuwai Hospital, from October 1, 2009, to September 30, 2012. We categorized the cohort into two groups based on the patients' secondary prevention of statin therapy before and after surgery: (1) continuation of statin therapy group (CS group), defined as patients who received statin therapy starting in the preoperative period and restarting early after surgery, and (2) discontinuation of statin therapy group (DS group), defined as those who received discontinuous statin therapy before or after surgery. This study was approved by the ethics committee at Fuwai Hospital. All the patients had previously granted permission for use of their medical records for research purposes.

### 2.2. Clinical Protocols and Surgical Techniques

CS group patients received moderate doses of either atorvastatin (20 mg), simvastatin (20-40 mg), or rosuvastatin (10 mg). All surgical procedures were performed by a single experienced cardiac surgeon (Dr. SUN), who had performed >6000 OPCAB procedures. A standard anaesthetic protocol was used throughout the study. All patients underwent a standard median sternotomy, where the internal mammary artery (left or right) and the saphenous vein were removed under direct vision. Single-vein grafts were used for patients with one distal anastomosis whenever possible. When sequential grafts were used, each graft had two anastomotic sites. No circular sequential grafts were used. No composite grafts were used. All anastomoses were hand-sutured. Coronary targets were stabilized in each patient with Octopus Evolution Tissue Stabilizer (Medtronic, Inc., Minneapolis, MN). Each bypass graft was assessed for transit-time flow measurement (Medistim Butterfly flowmeter, Medistim AS, Oslo, Norway) after anastomoses completion to obtain the mean flow values and pulsatile index.

### 2.3. Primary and Secondary Outcomes

Primary outcomes were graft patency at an average of five days after operation and in-hospital mortality. Secondary outcomes included intraoperative blood loss, liver, and renal functions.

### 2.4. Data Collection

Patients' demographics and clinical data were obtained from Fuwai Hospital Database. All patients underwent transthoracic echocardiography for the evaluation of left ventricular ejection fraction (LVEF). Blood samples were obtained to measure low-density lipoprotein (LDL) cholesterol, total cholesterol (TC), serum creatinine, alanine transaminase (ALT), aspartate transaminase (AST) concentrations, and so on.

### 2.5. Graft Patency by Multislice Computed Tomographic Angiography

Graft patency was documented by 64-slice multislice computed tomographic angiography (MSCTA) at discharge. All images were centrally stored and read by independent cardiovascular radiologists. A total of 582 patients were scheduled for systematic MSCTA at an average of five days after operation. The procedure was undertaken with a 64-slice MSCTA scanner (GE Healthcare, Milwaukee, WI). Images were transferred to a stand-alone workstation (Deep Blue, ADW4.3, GE Healthcare) and evaluated with dedicated analysis software. Scanned results were independently interpreted by two cardiovascular radiologists. Each graft was classified as patent (flow visible), occluded, or not analysable. Patency was defined as any flow through both the graft and the native vessel. The graft was determined to be nonpatent if either a stump was present or no flow could be observed.

### 2.6. Statistical Analysis

Inverse probability weighting (IPW) propensity-adjusted model is widely used for observational clinical studies to account for the covariates of treatment assignment bias [13, 14]. To neutralize the hidden and unmeasured confounders of treatment allocation bias, propensity scores were performed to balance continuation of statin (CS) and discontinuation of statin (DS) groups. These scores were incorporated in inverse probability weighted estimators to obtain that balance between the groups. To maximize pair-matching among the two groups, the propensity score included the following variables: core patient characteristics unevenly distributed among two groups before propensity matching. These variables included age, sex, body mass index (BMI), left ventricular ejection fractions (LVEF), left main disease or three-vessel disease, diabetes mellitus (DM), previous myocardial infarction (MI), stroke or transient ischemic attack (TIA), hypertension, smoking history, and current aspirin, beta-block, angiotensin-converting-enzyme inhibitor (ACEI), or angiotensin receptor blocker (ARB). Goodness-of-fit of this logistic regression model was appraised using the Hosmer-Lemeshow test (*P=*0.9918). If more than one CS group patients were matched to a DS group patient, the CS group patient was selected randomly among those patients. Ultimately, a well-matched cohort of patients was obtained, and early outcomes were compared between groups.

Continuous data were shown as mean. Student's t test was used to measure differences for variables with normal distribution and equal variances. Wilcoxon rank sum test was used for nonnormal distribution variables. Categorical data were displayed as frequencies and percentages, and comparisons were made with chi (*χ*)-square tests (Fisher exact tests if appropriate), with P values <0.05 being considered statistically significant. Odds ratios (OR) with 95% confidence interval (CI) of graft occlusion in response to statin therapy were derived from the Cox proportional hazards model, and their interaction *P* values were tested in subgroup analyses.

All statistical analyses were performed using SAS for Windows version 9.1 (SAS Institute, Cary, NC).

## 3. Results

### 3.1. Baseline Characteristics

A total of 648 consecutive patients underwent OPCAB by the same surgeon at Fuwai Hospital from October 1, 2009, to September 30, 2012. 66 patients were excluded, including atrial fibrillation in 24, renal dysfunction in 5, allergy to contrast agent in 5, patients' refusal in 16, and other medical problems in 16. Of the 582 patients included in the cohort analysis, 398 patients (68.4%) had received perioperative continuation of statin therapy (CS group), whereas the remaining 184 patients (31.6%) received discontinuous statin therapy as defined (DS group).

All were screened for graft patency assessment before discharge. The cohort included 457 men and 125 women, with median age of 61.5 years (range, 16-86 years). Among the 582 patients undergoing OPCAB, a significant history of hypertension (68.8%), diabetes (36.9%), previous myocardial infarction (34.5%), and current smoking (51.6%) was observed. Furthermore, 225 patients (38.6%) had left main disease, and 502 patients (86.3%) had three-vessel disease. Mean distal anastomoses were 3.2 (range, 1-6 grafts) per patient. With respect to medication, 69% of the patients took continuous aspirin therapy (100 mg daily) perioperatively, and 88% received beta-blockers after surgery.

The pre- and postinverse probability weighting (IPW) adjusted baseline characteristics of patients are presented according to the statin treatment strategies in Tables 1 and 2. In an IPW propensity-adjusted model, all the clinical baseline characteristics were similar between the two groups (including age, gender, BMI, left main disease or triple vessel disease, LVEF, diabetes, smoking, hypertension, previous MI, stroke or TIA, medication of aspirin, beta-blocker, and ACEI or ABR). The mean graft flow values and pulsatile index by transit-time flow measurement were shown in Table 3.

### 3.2. Primary Outcomes following OPCAB

Neither group had any deaths following OPCAB. Overall patency rate was 98.29% (1838 of 1870 grafts), at an average of five days after operation. Among patients who underwent OPCAB, early graft patency rates were 98.4% (1255 of 1275 grafts) in the CS group and 98.0% (583 of 595 grafts,* P*=0.486) in the DS group before IPW adjustment. This lack of significant difference in graft patency between CS and DS groups also held true following IPW adjustment (98.5% versus 98.0%,* P=*0.224), suggesting that perioperative continuation of statin therapy was not associated with a significant graft patency benefit (Table 4).

### 3.3. LDL Cholesterol

Both LDL and TC were reduced from baseline in both groups, with the resulting postdischarge levels being significantly lower in the CS than in DS group (LDL 65.35 mg/dL versus 76.57 mg/dL,* P*=0.038; TC 3.33 mmol/L versus 3.56 mmol/L,* P*<0.001) before IPW adjustment. This significant difference between groups was observed following IPW adjustment (LDL 65.02 mg/dL versus 78.56 mg/dL; TC 3.32 mmol/L versus 3.63 mmol/L, both* P*<0.001) (Table 5). Low-density lipoprotein (LDL) levels of patients on continuous statin treatment at discharge were under the target level of aggressive LDL lowering therapy (70 mg/dL).

### 3.4. Liver and Renal Functions

Following IPW adjustment, ALT levels were well balanced at baseline but increased to a significantly greater extent among DS patients than among CS ones at discharge (49.67 U/L versus 34.52 U/L,* P*<0.001). AST levels were similar at baseline but higher in CS group than in DS group at discharge (33.54 U/L versus 28.10 U/L,* P*<0.001). Clinically relevant ALT (or AST) elevation is defined as an increase of three times the upper limit of normal. Among patients who underwent OPCAB, in an IPW propensity-adjusted model, abnormal ALT elevation was observed in 8.9% of the CS group and 3.1% in the DS group (OR 3.06, 95% CI, 1.77 to 5.29,* P<*0.001), suggesting that perioperative continuation of statin therapy was associated with elevated liver function. Serum creatinine levels at discharge were similar between groups after IPW adjustment (1.04 mg/dL versus 1.06 mg/dL,* P=*0.331). No significant differences were present between CS and DS groups regarding estimated glomerular filtration rate (GFR) (76.28 mL/min/1.73m^2^ versus 76.13 mL/min/1.73m^2^,* P=*0.90).

### 3.5. Intraoperative Blood Loss

In the entire cohort, intraoperative blood loss was significantly greater in the DS than in CS group before (432.46 mL versus 521.23 mL,* P*=0.001) and following IPW adjustment (438.53 mL versus 480.47 mL,* P=*0.010) (Table 5).

### 3.6. Subgroup Analyses for Graft Occlusion

**Figure 1:**
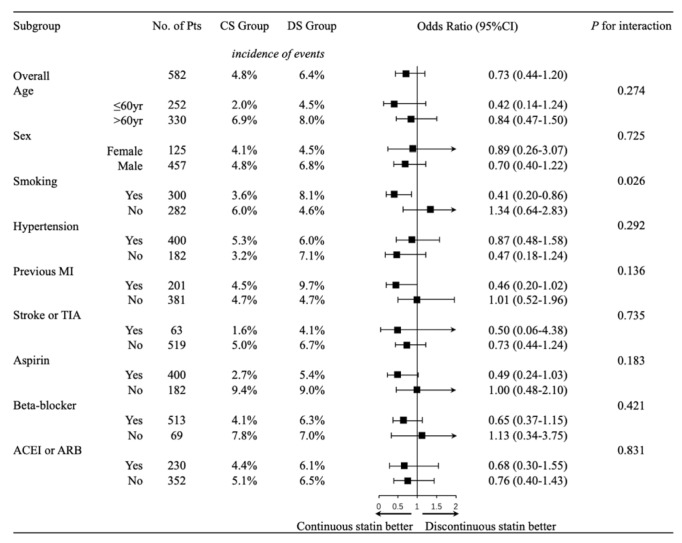
*Effect of Perioperative Statin Therapy on Postoperative Graft Occlusion, Overall and According to Prespecified Subgroups Following IPW Adjustment*. Shown are odds ratios for the incidence of graft occlusion after off-pump CABG (assessed by MSCTA) among patients with perioperative continuation of statin therapy (CS group) as compared with those with discontinuation of statin therapy (DS group). For each prespecified subgroup, after IPW propensity-score adjustment, squares represent odds ratios and horizontal lines represent 95% confidence intervals, with values and results of the *P* for interaction significance test presented alongside. A square to the left of the vertical line indicates a benefit associated with perioperative continuous statin use, but the benefit is significant at the 5% level only if the horizontal line does not overlap the vertical line.

Within the prespecified subgroups, there was no significant difference between CS and DS groups following IPW adjustment, regarding graft occlusion with respect to age (both ≤60 and >60 year), sex, aspirin treatment, hypertension, DM, previous MI, stroke or TIA, current medication of beta-blocker, ACEI, or ARB. However, following IPW adjustment, perioperative continuous statin therapy was associated with more than 50% reduction in graft occlusion rate among smoking patients (3.6% versus 8.1%, OR 0.41; 95% CI, 0.20 to 0.86;* P*<0.05), a difference that was absent in the nonsmokers (6.0% versus 4.6%, OR 1.34; 95% CI, 0.64 to 2.83). Furthermore, our finding indicated that there was a significant interactive effect of smoking and continuous perioperative statin treatment on graft occlusion (*P*-interaction = 0.026). Perioperative continuation of statin therapy might reduce the graft occlusion rate in smoking population, compared to discontinuous statin therapy (Figure 1).

## 4. Discussion

In this retrospective, propensity-score matched cohort study, our findings showed that the early graft patency did not differ between patients receiving perioperative continuous statin therapy and those receiving discontinuous statin therapy, both before and after IPW adjustment. Lower risk of graft occlusion among smoking patients was observed for continuous statin therapy group compared to discontinuous statin group. In addition, patients prescribed a continuous statin therapy correlated with an excess of abnormal liver function.

### 4.1. Statin Treatment and Graft Patency

The absence of a significant difference in graft-patency rate may be due in part to the following reasons. (1) Moderate statin dose and various statins: patients in the cohort were prescribed different types of statins, including atorvastatin, simvastatin, or rosuvastatin. Despite a possible difference in pleiotropic effects in various statins [15], limited data showed that a certain statin type was associated with beneficial effects on graft-patency, and current available evidence suggests that the clinical benefit is largely independent of the type of statin but depends on the extent of LDL-C lowering. It should be noted that mean LDL levels of patients on continuous statin treatment were reduced to 65.35 mg/dL (before IPW adjustment) and 65.02 mg/dL (after IPW adjustment), both of which were lower than the target level of aggressive LDL lowering therapy (70 mg/dL), as established by the 2018 AHA/ACC guideline [16]. (2) Short exertion duration: we performed MSCTA for graft patency assessment before discharge at an average of five days after operation. Thus, it is likely that a longer period of statin therapy may be needed before visible effects on graft patency can be observed. This possibility, however, should be balanced by the fact that antioxidant and anti-inflammatory effects develop rapidly after initiating statin therapy [17, 18]. To sum up, the primary outcome did not show any beneficial effect of perioperative continuation of statin therapy on early graft patency.

### 4.2. Secondary Outcomes

#### 4.2.1. Liver Function

Liver function elevation is one of the most common statin-associated side effects. The activity of ALT in plasma is commonly used to assess hepatocellular damage. In this study, we found that both ALT and AST levels increased after perioperative continuation of statin therapy. Also, we found that the abnormal ALT elevation rate, a key indication of abnormal liver function, was significantly higher in the CS group. Our finding regarding clinically relevant ALT elevation was consistent with findings reported by other studies suggesting stains being frequently associated with elevated liver functions. Additionally, elevated ALT incidence in this cohort is higher than that in other studies, where clinical trials there had a relative low incidence of “statin-associated symptoms.” This could be explained by the mechanism that Asians have more side effects from drugs than do non-Asians [19]. Specifically, plasma exposure to rosuvastatin, one of the most common used statins, and its metabolites, is significantly higher for a given dose in Asian persons especially in Chinese population than in whites [20].

#### 4.2.2. Renal Function

There is limited data about long-term renal function benefit related to perioperative statin use among OPCAB populations, although the TNT clinical trial showed that intensive lipid lowering treatment could improve GFR in coronary heart disease patients [21]. On the contrary, a randomized trial enrolling 1922 patients scheduled for cardiac surgery found that acute kidney injury was more common with perioperative rosuvastatin use [2]. In our study, the GFR did not show any difference between the CS and DS groups. This result may due to the complex types of statins in the cohort and the short duration of perioperative statin therapy.

#### 4.2.3. Other Outcomes

Intraoperative blood loss was significantly higher in DS than in the CS group. Rapid anti-inflammatory effect may contribute to the lower incidence in CS, but the role of perioperative statin use in reducing blood loss is still unknown.

### 4.3. Subgroup Analyses

In our study, we showed that there was a significant interactive effect of smoking and continuous perioperative statin treatment on graft occlusion (*P* = 0.026). Our finding, if confirmed, suggests that smokers scheduled to undergo OPCAB may particularly benefit from perioperative continuation of statin treatment to prevent graft occlusion. Widely recognized as one of the risk factors of coronary artery disease, smoking affects not only the development of saphenous vein graft (SVG) failure [22, 23], but also artery graft atheroma and thus decreased graft patency [24]. Statin has been shown to have rapid anti-inflammatory and antioxidant effects independent of their effect on low density lipoprotein cholesterol [25]. Recently, a meta-analysis study, including 11 trials and 89604 individuals, showed that statins may ease smoking-caused inflammation [26]. In our study, perioperative continuation of statin therapy reduces graft occlusion rate by more than half among smoking patients. This result, showing that smokers might benefit more from continuous statin treatment compared to nonsmokers, may be explained by the rapid developments of antioxidant and anti-inflammatory effects after statin initiation.

The biological mechanism by which perioperative continuation of statin therapy can prevent graft occlusion in smoking patients remains to be determined. The beneficial effect of continuous statin use may stem from its multiple anti-inflammatory effects. For example, cigarette smoke-induced atherosclerosis involves several systemic pathways and underlying mechanisms [27], which are still not completely understood. Endothelial dysfunction caused by smoking is associated with decreased endothelial nitric oxide synthase (eNOS) expression [28–30].

Statin might effectively decrease the adverse impact of smoking with its anti-inflammatory, antioxidant, and antithrombotic effects [31–33] or have direct interactions with eNOS, which may partially counteract the adverse effect of smoking on the development of graft failure. But how it transfers to the reduction of graft failure has been not clearly known.

The notion of perioperative statin therapy being associated with beneficial effects on in-hospital clinical outcomes among cardiac surgery was not universally endorsed, as reflected in the title of one commentary: “Do not STICS to statins in cardiac surgery” [34]. Some thoughtful critics felt that it was the matter of race and timing that led to the lack of evidence of beneficial effects of short-term statin therapy [35]. Proponents argued that antioxidant and anti-inflammatory effects developed rapidly after the initiation of statin therapy. 2015 AHA guidelines recommended that all CABG patients should receive statin therapy, starting in the preoperative period and restarting early after surgery unless contraindicated [36], with the aggressive LDL target level just over the horizon. 2017 EACTS guidelines were skeptical [1] because data showed that efficacy and safety of perioperative statin use still remained controversial. This open debate helped to shape the better design of perioperative statin strategy for CABG populations. In the end, however, the questions could only be answered with evidence-based data.

In summary, perioperative continuous statin use still remains suboptimal despite beneficial effects among smoking patients.

### 4.4. Potential Limitation

In view of limitations of the observational study, an IPW propensity-adjusted model was conducted to neutralize the confounders of treatment assignment bias. All operations were performed by a single experienced surgeon to diminish surgical technical bias. The lack of overall patency benefit of perioperative statin therapy might be related to moderate-intensity statin use. Long-term treatment of statin contributes to the stabilization of plaques. In this study, CS group showed 100% of preoperative statin use while it was only 28.3% in DS group. Multicentre studies and long-term follow-up are required to prove the efficacy and safety of continuous statin therapy in off-pump CABG.

## 5. Conclusions

Among patients undergoing OPCAB, perioperative continuation of statin therapy did not improve early graft patency while subgroup analyses showed that smoking patients may benefit from perioperative continuous statin therapy in terms of graft occlusion. Additionally, perioperative continuous statin use tended to increase the risk of elevated liver function.

## Figures and Tables

**Table 1:** Baseline and graft characteristics of overall patient before IPW Adjustment.

Patient baseline and graft characteristics	CS group	DS group	*P*-Value
	(n=398)	(n=184)	
Age, years	62.1	60.2	0.038
≤60yr, %	40.5	49.5	0.042
>60yr, %	59.5	50.5	0.042
Female, %	22.9	18.5	0.230
BMI, kg/m^2^	25.3	26.0	0.007
Current smoking, %	50.8	53.3	0.570
Diabetes mellitus, %	37.9	34.8	0.460
Hypertension, %	72.4	60.9	0.005
Dyslipidemia, %	68.8	58.5	0.014
Acute coronary syndrome, %	72.1	66.3	0.160
Left main disease, %	38.4	39.1	0.870
Triple vessel disease, %	86.7	85.3	0.660
Previous PCI, %	16.8	8.2	0.002
Previous MI, %	36.7	29.9	0.110
Stroke or TIA, %	11.6	9.2	0.400
LVEF, %	60.3	59.8	0.540
LDL-C, mg/dL	96.67	93.97	0.410
TC, mmol/L	4.35	4.19	0.090
Creatinine, mg/dL	0.94	0.94	0.850
GFR, mL/min/1.73m^2^ †	82.27	84.58	0.120
ALT, U/L	29.17	36.51	0.004
AST, U/L	22.08	23.63	0.170
Medication			
Aspirin, %	64.1	78.8	<0.001
Beta-blocker, %	88.7	87.0	0.550
ACEI or ARB, %	48.0	21.2	<0.001
Distal anastomoses (mean distal)	1275 (3.2)	595 (3.2)	0.683
Total graft	1215	561	0.952
SVG	831	378	0.614
Arterial graft	384	183	0.130
Sequential bypass anastomoses	60	34	0.312

IPW = inverse probability weighting; CS = perioperative continuation of statin therapy; DS = discontinuous statin therapy; IHD = ischemic heart disease; PCI = percutaneous coronary intervention; MI = myocardial infarction; TIA = transient ischemic attack; LVEF = left ventricular ejection fraction; LDL-C = low-density lipoprotein cholesterol; TC = total cholesterol; GFR = glomerular filtration rate; ALT = alanine transaminase; AST = aspartate aminotransferase; ACEI = angiotensin-converting-enzyme inhibitor; ARB = angiotensin receptor blocker; SVG = saphenous vein graft.

†The glomerular filtration rate (GFR) was estimated with the Chronic Kidney Disease Epidemiology Collaboration (CKD-EPI) equation.

**Table 2:** Baseline characteristics of overall patient after IPW Adjustment.

Patient baseline characteristics	CS group	DS group	*P*-Value
Age, years	61.4	62.2	NS
≤60yr, %	43.2	46.0	NS
>60yr, %	56.8	54.0	NS
Female, %	20.9	19.3	NS
BMI, kg/m^2^	25.5	25.4	NS
Current smoking, %	51.9	51.3	NS
Diabetes mellitus, %	38.1	39.2	NS
Hypertension, %	67.9	68.5	NS
Dyslipidemia, %	69.1	64.0	NS
Acute coronary syndrome, %	71.7	67.5	NS
Left main disease, %	37.8	38.9	NS
Triple vessel disease, %	86.0	87.3	NS
Previous MI, %	34.5	33.6	NS
Stroke or TIA, %	10.6	12.6	NS
LVEF, %	60.1	60.4	NS
LDL-C, mg/dL	95.98	98.30	NS
TC, mmol/L	4.33	4.33	NS
Creatinine, mg/dL	0.939	0.937	NS
GFR, mL/min/1.73m^2^	83.25	83.76	NS
ALT, U/L	31.44	31.19	NS
AST, U/L	22.54	22.27	NS
Medication			
Aspirin, %	69.2	73.3	NS
Beta-blocker, %	86.7	87.6	NS
ACEI or ARB, %	39.1	39.3	NS

**Table 3:** Transit-time flow outcomes: mean graft flow and pulsation index in CS and DS groups.

Graft position	Graft flow, mL/min			Pulsation index		
	CS group	DS group	*P*-Value	CS group	DS group	*P*-Value
LIMA: anterior wall	22.80±15.94	22.20±11.31	0.66	2.26±0.64	2.28±0.60	0.76
Aorta: anterior wall	46.62±27.34	48.60±23.31	0.55	1.87±0.67	1.82±0.38	0.55
Aorta: lateral wall	40.41±21.02	44.43±22.37	0.06	2.05±0.95	1.94±0.74	0.22
Aorta: inferior wall	43.63±23.07	45.06±24.73	0.59	1.80±0.67	1.93±0.93	0.13
Occluded graft	29.10±19.57	35.08±23.38	0.44	2.10±0.63	2.61±1.17	0.18

LIMA = left internal mammary artery.

**Table 4:** Primary outcome: effect on early graft patency.

Graft patency	Before IPW adjustment			After IPW adjustment		
	CS group (n=398)	DS group (n=184)	*P*-Value	CS group	DS group	*P*-Value
Patency of all grafts, %	98.4	98.0	0.49	98.5	98.0	0.22
	(1255/1275)	(583/595)				
Patency of all patients, %	95.2	94.0	0.54	95.2	93.6	0.24
	(379/398)	(173/184)				

**Table 5:** Secondary outcomes: effect on liver function and clinical outcomes at discharge.

Secondary outcomes	Before IPW Adjustment			After IPW Adjustment		
	CS group (n=398)	DS group (n=184)	*P*-Value	CS group	DS group	*P*-Value
LDL-C, mg/dL	65.35	76.57	0.038	65.02	78.56	<0.001
TC, mmol/L	3.33	3.56	<0.001	3.32	3.63	<0.001
Creatinine, mg/dL	1.04	1.05	NS	1.04	1.06	NS
GFR, mL/min/1.73m^2^ †	75.53	77.46	NS	76.28	76.13	NS
ALT, U/L	48.51	36.56	0.001	49.67	34.52	<0.001
AST, U/L	33.20	27.97	0.017	33.54	28.10	<0.001
Abnormal ALT, % (n) ‡	8.5 (34)	3.8 (7)	0.038	8.9	3.1	<0.001
Abnormal AST, % (n) ‡	1.3 (5)	1.1 (2)	NS	1.2	1.0	NS
Intraoperative blood loss, mL	432.46	521.23	0.001	438.53	480.47	0.010

LDL-C = low-density lipoprotein cholesterol; TC = total cholesterol; GFR = glomerular filtration rate; ALT = alanine transaminase; AST = aspartate aminotransferase.

†The glomerular filtration rate (GFR) was estimated with the Chronic Kidney Disease Epidemiology Collaboration (CKD-EPI) equation.

‡Abnormal ALT (or AST) was defined as ALT (or AST) >3×upper limit of normal (ULN).

## Data Availability

The data used to support the findings of this study are available from the corresponding author upon request.

## Supplementary Materials

Supplementary Materials Table 1: Timing of Statin Discontinuation. The timing of statin discontinuation: patients were classified into 2 groups: patients receiving both preoperative statin therapy at admission and restarting early after OPCAB (CS group, n=398) and patients discontinuing statin therapy preoperatively or postoperatively (DS group, n=184). CS = perioperative continuation of statin therapy; DS = discontinuous statin therapy; POD=postoperative day *∗*Starting before surgery and restarting early after surgery. Table 2: Graft Characteristics of Overall Patient. The number of distal anastomoses and different graft types are summarized. SVG = saphenous vein graft; LIMA = left internal mammary artery; RIMA = right internal mammary artery; RA = radial artery. Table 3: Transit-time Flow Outcomes, Mean Graft Flow and Pulsation Index in CS and DS Groups. The graft flow and pulsation index by transit-time flow measurement were similar between groups. LIMA = left internal mammary artery
