# Supplementary material for: Does Early Graft Patency Benefit from Perioperative Statin Therapy? A Propensity Score-Matched Study of Patients Undergoing Off-Pump Coronary Artery Bypass Surgery
Source: Cardiovasc Ther. 2019 Aug 6;2019:1582183. doi: 10.1155/2019/1582183 (PMC6739783; doi:10.1155/2019/1582183)
Supplement: Supplementary Materials — Table 1: Timing of Statin Discontinuation. The timing of statin discontinuation: patients were classified into 2 groups: patients receiving both preoperative statin therapy at admission and restarting early after OPCAB (CS group, n=398) and patients discontinuing statin therapy preoperatively or postoperatively (DS group, n=184). CS = perioperative continuation of statin therapy; DS = discontinuous statin therapy; POD=postoperative day ∗Starting before surgery and restarting early after surgery. Table 2: Graft Characteristics of Overall Patient. The number of distal anastomoses and different graft types are summarized. SVG = saphenous vein graft; LIMA = left internal mammary artery; RIMA = right internal mammary artery; RA = radial artery. Table 3: Transit-time Flow Outcomes, Mean Graft Flow and Pulsation Index in CS and DS Groups. The graft flow and pulsation index by transit-time flow measurement were similar between groups. LIMA = left internal mammary artery [file 1582183.f1.docx]

# Supplementary Materials

**Table 1. Timing of Statin Discontinuation**

| Statin duration | Discontinued timing | CS Group (n=398) | DS Group (n=184) |
| --- | --- | --- | --- |
| Admission - discharge* | NA | 398 | 0 |
| Admission - surgery | After surgery | 0 | 52 |
| POD1 - discharge | Admission | 0 | 1 |
| POD2 - discharge | Admission | 0 | 40 |
| POD3 - discharge | Admission | 0 | 20 |
| POD4 - discharge | Admission | 0 | 19 |
| POD5 - discharge | Admission | 0 | 17 |
| POD6 - discharge | Admission | 0 | 8 |
| POD7 or later - discharge | Admission | 0 | 7 |
| Started after surgery and stopped at least 1 day before discharge | Admission | 0 | 20 |

The timing of statin discontinuation: Patients were classified into 2 groups: patients receiving both preoperative statin therapy at admission and restarting early after OPCAB (CS group, n=398) and patients discontinuing statin therapy preoperatively or postoperatively (DS group, n=184).

CS = perioperative continuation of statin therapy; DS = discontinuous statin therapy; POD=postoperative day

*Starting before surgery and restarting early after surgery.

**Table 2. Graft Characteristics of Overall Patient**

| Graft Characteristics | CS Group  (n=398) | DS Group  (n=184) | *P*-Value |
| --- | --- | --- | --- |
| Distal anastomoses (mean distals) | 1275 (3.2) | 595 (3.2) | 0.683 |
| Total graft | 1215 | 561 | 0.952 |
| SVG | 831 | 378 | 0.614 |
| Arterial graft | 384 | 183 | 0.130 |
| LIMA | 376 | 180 | 0.069 |
| RIMA | 6 | 3 | 0.911 |
| RA | 2 | 0 | 0.336 |
| Sequential bypass anastomoses | 60 | 34 | 0.312 |

The number of distal anastomoses and different graft types are summarized.

SVG = Saphenous vein graft; LIMA = Left internal mammary artery; RIMA = Right internal mammary artery; RA = Radial artery

**Table 3. Transit-time Flow Outcomes: Mean Graft Flow and Pulsation Index in CS and DS Groups**

| Graft Position | Graft Flow, mL/min | | |  | | Pulsation Index | | | |
| --- | --- | --- | --- | --- | --- | --- | --- | --- | --- |
|  | CS Group | DS Group | *P*-Value | |  | | CS Group | DS Group | *P*-Value |
| LIMA - anteior wall | 22.80±15.94 | 22.20±11.31 | 0.656 | |  | | 2.26±0.64 | 2.28±0.60 | 0.756 |
| Aorta - anterior wall | 46.62±27.34 | 48.60±23.31 | 0.549 | |  | | 1.87±0.67 | 1.82±0.38 | 0.553 |
| Aorta - lateral wall | 40.41±21.02 | 44.43±22.37 | 0.064 | |  | | 2.05±0.95 | 1.94±0.74 | 0.222 |
| Aorta - inferior wall | 43.63±23.07 | 45.06±24.73 | 0.588 | |  | | 1.80±0.67 | 1.93±0.93 | 0.127 |
| Occluded Graft | 29.10±19.57 | 35.08±23.38 | 0.442 | |  | | 2.10±0.63 | 2.61±1.17 | 0.181 |

The graft flow and pulsation index by transit-time flow measurement were similar between groups.

LIMA = Left internal mammary artery
